# *GLI1*-Rearranged Enteric Tumors: Updates on Clinicopathologic and Molecular Genetics Features

**DOI:** 10.3390/cells14020118

**Published:** 2025-01-14

**Authors:** Ahmed I. Younes, Haider A. Mejbel

**Affiliations:** Department of Pathology and Laboratory Medicine, Emory University School of Medicine, Atlanta, GA 30322, USA; ahmed.younes@emory.edu

**Keywords:** *GLI1*, fusion gene, next-generation sequencing, gastrointestinal tumors

## Abstract

Recent advances in molecular genetics, particularly in identifying and characterizing genetic abnormalities within mesenchymal neoplasms, have led to a more comprehensive and evolving classification system. Modern technological developments in cytogenetics and next-generation sequencing have enabled the analysis of small clinical samples, expanded our understanding of tumor biology, and improved the diagnostic, prognostic, and predictive precision by identifying targeted genetic alterations, confirming the presence of fusion transcripts, and/or revealing the overexpression of specific genes and their targets. In this review, we focus specifically on the *GLI1*-rearranged enteric tumor, a recent clinicopathological entity that has emerged within the expanding classification of mesenchymal tumors. Herein, we aim to explore the histopathological features, molecular genetic characteristics, and clinical outcomes in these tumors. Due to their rarity and the extensive overlapping in their histopathological and molecular features with other neoplasms, continued research and systematic documentation of *GLI1*-rearranged enteric tumors is necessary to better understand their biological behavior, develop more accurate prognostic indicators, and establish optimal treatment strategies.

## 1. Clinical and Epidemiologic Characteristics of *GLI1*-Rearranged Enteric Tumors

**Figure 1:**
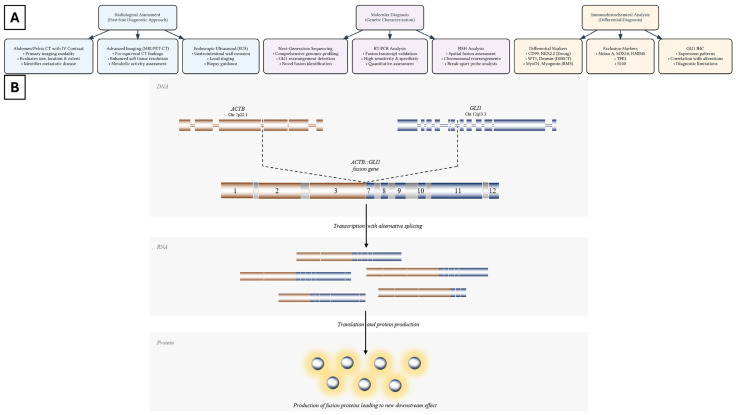
(**A**) Schematic diagram illustrating the diagnostic workflow and *GLI1* rearrangement mechanism. (**B**) The fusion gene is formed by the fusion of the promoter region of *ACTB* exon 3 juxtaposing the coding region of *GLI1* exon 7. Transcription with alternative splicing results in multiple chimeric isoforms that undergo translation to increase GLI1 protein production and overexpression and ultimately create a new downstream effect.

GLI1-rearranged enteric tumors are a newly proposed category of mesenchymal neoplasms, most commonly found in the gastrointestinal tract, and are defined by rearranging the *GLI1* gene at chromosome 12q13.3 with various pathogenic fusion partners, most commonly ACTB::GLI1 and MALAT1::GLI1 [1]. These tumors affect individuals with a wide age distribution, ranging from 2 to 71 years, with almost equal sex distribution [1,2]. Within the gastrointestinal tract, these tumors typically arise in the stomach and small intestine, and most are localized at the submucosal and muscularis propria compartments, with an average tumor size of approximately 5 cm [1,2]. Based on the tumor size and anatomic location, the clinical presentations of GLI1-rearranged enteric tumors may vary, ranging from being asymptomatic and incidentally detected to abdominal pain, alterations in bowel habits, and delayed gastric emptying secondary to mass effect [1,3]. GLI1-rearranged enteric tumors are usually indolent; however, tumors ≥ 6 cm are considered high-risk due to their potential for local invasion and distant metastasis to other organs such as the liver, soft tissue, brain, and lung [4,5,6]. On imaging, GLI1-rearranged enteric tumors usually appear as clearly demarcated neoplasms arising from the affected segment of the gastrointestinal wall [1]. Abdomen/pelvis computed tomography (CT) with intravenous contrast provides valuable information about the tumor’s size, location, and relationship to other anatomical organs; the extent of the primary tumor; and the presence of any metastatic disease (Figure 1A). Other modalities such as magnetic resonance imaging and positron emission tomography–CT can be particularly useful when CT is equivocal or when there is a suspicion of distant metastases (Figure 1A) [7,8]. In contrast, endoscopic ultrasound can be used to evaluate the depth of tumor invasion within the gastrointestinal wall (Figure 1A) [9].

## 2. Histopathologic and Immunophenotypic Characteristics of *GLI1*-Rearranged Enteric Tumors

GLI1-rearranged enteric tumors present as well-circumscribed lesions, composed of distinctive epithelioid cells and, to a lesser extent, spindle cells with varying degrees of mitotic activity, usually less than 2/10 HPFs [10]. The architectural pattern of these tumors is notable for their arrangements of nests, cords, and/or fascicles, all of which are notably associated with a prominent capillary vascular network. In addition, the tumor cells display a characteristic clear-to-eosinophilic cytoplasm, uniform round nuclei containing fine chromatin patterns, and subtle, often inconspicuous nucleoli [1,5]. Prior studies have highlighted key histological parameters, such as increased mitotic activity (≥4 mitoses per 10 high-power fields), the presence of necrosis, and a tumor size of 5 cm or greater, all of which are shown to be associated with poor clinical outcomes [4].

The immunohistochemical profile of GLI1-rearranged enteric tumors is distinctive, showing strong positivity for S100, while consistently lacking expression of pancytokeratin, muscle-specific actin, desmin, CD117 (C-KIT), DOG1, SOX10, B-catenin, MyoD1, myogenin, STAT6, CD34, ERG, D2-40, WT1, calretinin, BCOR, ALK1, cathepsin K, and NUT (Figure 1A). INI1 and BRG1, surrogate markers that are typically used to evaluate for SMARCB1- and SMARA4-deficient tumors, are retained within the tumor cells. GLI1-rearranged enteric tumors may show a focal-to-patchy expression of synaptophysin and CD56, which may pose a diagnostic pitfall by mimicking neuroendocrine tumors [1,6,11]. The diagnostic application of GLI1, as well p16 immunohistochemical staining, was analyzed in a cohort of GLI1-altered neoplasms, comprising equal numbers of GLI1-amplified and GLI1-rearranged tumors. The immunostaining patterns revealed distinctive characteristics: both GLI1-amplified and GLI1-rearranged tumors consistently demonstrated robust immunoreactivity for GLI1 immunohistochemical staining, with a notable and distinctive pattern between the two molecular subtypes. The GLI1-amplified cases exhibited a predominant nuclear staining pattern, while the GLI1-rearranged cases showed a characteristic cytoplasmic distribution of the GLI1 protein. A particularly significant finding emerged in the p16 expression analysis, where strong-to-moderate immunoreactivity was exclusively observed in GLI1-amplified tumors, establishing a crucial diagnostic marker that can help distinguish between the two molecular subtypes. This differential staining pattern of GLI1 immunohistochemical stain proved highly reliable, achieving perfect sensitivity (100%) and near-perfect specificity (93%) in identifying GLI1-amplified cases. The dual GLI1/p16 immunohistochemical panel thus emerges as a cost-effective and practical screening approach, enabling pathologists to effectively identify and distinguish GLI1-altered neoplasms and potentially guiding subsequent molecular testing and therapeutic decisions [12].

Nonetheless, the diagnosis of GLI1-rearranged enteric tumors can be a significant challenge because of their overlapping clinical, morphological, and immunophenotypic characteristics with various other neoplasms, leading to a broad differential diagnosis [1]. Due to their lobulated architecture and nested growth pattern, GLI1-rearranged enteric tumors may mimic myoepithelial neoplasms and pseudoendocrine sarcomas [13]. This might be further complicated when these tumors also exhibit a round-to-ovoid cell morphology, clear cytoplasm, and myxoid stroma, as well as showing a positive expression for S100 [1,2]. However, several distinguishing features can aid in the identification of GLI1-rearranged enteric tumors, such as the presence of a well-developed arborizing vascular network between the tumor nests, in conjunction with the lack of positive expression for myoepithelial markers, such as P63, glial fibrillary acidic protein (GFAP), and beta-catenin [1,13]. In cases where the morphological and immunohistochemical findings are inconclusive, molecular testing emerges as a crucial diagnostic tool, as myoepithelial tumors typically harbor *EWSR* or *FUS* rearrangements [14].

Another important differential diagnostic consideration includes pericytic (perivascular) tumors, such as cellular glomus tumors and/or cellular myopericytoma. These tumors may display overlapping morphologic features with GLI1-rearranged enteric tumors, including an epithelioid cell morphology with generalized uniformity, and scattered mitotic activity with lobular or solid growth patterns. Although the positive expression of SMA and negative expression of S100 can help support the diagnosis, the presence of recurrent *NOTCH* and *SRF* gene rearrangements in pericytic tumors (glomus tumors and cellular myopericytoma, respectively) provides a critical diagnostic tool for differentiation from *GLI1*-rearranged enteric tumors [15]. It is important to note that there are considerably different viewpoints that persist regarding the precise classification of GLI1-rearranged enteric tumors and their relationship to myopericytic/pericytic tumors. Some authors still classify these tumors as t (7;12) pericytomas, while others contest the pericytic nature of these lesions. This ongoing discussion highlights the need for further research to better define their biological characteristics and establish a consensus classification [16,17].

GLI1-rearranged enteric tumors, in addition to a low-grade uniform cytomorphology and lobulated growth pattern, may exhibit a positive expression of synaptophysin and therefore mimic well-differentiated neuroendocrine tumors (WDNWTs) and paraganglioma, both histologically and by immunohistochemistry. However, both WDNETs and paraganglioma also express chromogranin, and the cytokeratin expression in WDNWTs and GATA3 expression in paraganglioma also help differentiate these tumors from GLI1-rearranged enteric tumors [1,18]. On the other hand, GLI1-rearranged enteric tumors may occasionally exhibit high-grade features including a high nuclear-to-cytoplasmic ratio, brisk mitotic activity, and tumor necrosis, with small as well as rhabdoid cell morphologies that may overlap with small, round, and blue cell neoplasms including Ewing sarcoma, CIC-rearranged sarcoma, desmoplastic small round blue cell tumor (DSRBCT), and rhabdomyosarcoma. However, these tumors can generally be distinguished from these histologic mimics by a combination of immunohistochemistry and molecular testing. The strong and diffuse expression of membranous CD99 and positivity for NKX2.2 favors the diagnosis of Ewing sarcoma, which will typically be negative in GLI1-rearranged enteric tumors [19]. Similarly, the strong positivity for WT1 carboxy-terminus and dot-like expression of desmin favor the diagnosis of DSRBCTs, as well as CIC-rearranged sarcoma [20]. Immunohistochemistry for MyoD1 and myogenin can be performed to exclude rhabdomyosarcoma (Figure 1A) [21]. Likewise, while alveolar soft part sarcoma shares certain architectural patterns with GLI1-rearranged enteric tumors, including nested growth and vascular prominence, it demonstrates distinctive cytological and immunophenotypic features, characterized by large polygonal cells with an abundant eosinophilic cytoplasm, conspicuous nucleoli, and positive immunoreactivity for both PAS and TFE3 [22]. Due to S100 immunoreactivity, tumors such as melanoma with epithelioid morphologies should be considered in the differential diagnosis of GLI1-rearranged enteric tumors. Therefore, additional immunohistochemical studies such as SOX10, Melan A, and HMB45 can be performed to distinguish melanoma from GLI1-rearranged enteric tumors [23].

Although GLI1 immunohistochemical staining can help evaluate for GLI1-rearranged enteric tumors, GLI1 expression is not entirely specific, as it is also noted in a subset of other tumor types with secondary *GLI1* copy number gains, such as dedifferentiated liposarcomas, leiomyosarcomas, and pleomorphic liposarcomas. Therefore, a comprehensive diagnostic approach incorporating morphological, immunohistochemical, and molecular features is still needed when interpreting GLI1 immunostaining results [24]. Nonetheless, advanced molecular diagnostic methodologies can serve as an essential confirmatory tool for precise tumor classification. For instance, confirmatory molecular testing for an *EWSR1* rearrangement could be performed when the histopathological findings overlap with Ewing sarcoma [25], and *NR4A3* gene fusion serves for cases that may overlap with extraskeletal myxoid chondrosarcoma [26]. Nonetheless, in addition to arriving at an accurate diagnosis, molecular characterization may provide helpful prognostic information and identify potential therapeutic targets for GLI1-rearranged enteric tumors.

## 3. Molecular Characteristics of *GLI1*-Rearranged Enteric Tumors

Tumors with *GLI1* alterations are characterized by either *GLI1* gene fusions or amplifications, both of which lead to abnormal activation of *GLI1* [10]. The activation of *GLI1* in neoplastic cells is associated with increased cell proliferation, angiogenesis, and resistance to treatment [27]. Common variants associated with *GLI1* fusions include MALAT1::GLI1 and ACTB::GLI1 gene fusion (Figure 1B) [28,29]. The MALAT1::GLI1 fusion gene has emerged as particularly intriguing due to its presence in two distinctly different gastric neoplasms [5,28]. The first of these is plexiform fibromyxoma, which is a rare gastric tumor that typically develops in the pyloric region and exhibits a unique multinodular and plexiform architecture [28]. Under microscopic examination, these tumors show a homogeneous spindle cell population and consistently demonstrate SMA positivity. Despite sharing molecular similarities with GLI1-rearranged enteric tumors, plexiform fibromyxoma maintains its status as a distinct clinicopathologic entity due to its unique biological behavior and clinical presentation. The second tumor type harboring the MALAT1::GLI1 fusion is gastroblastoma, which presents with markedly different characteristics. These neoplasms typically arise in the gastric antrum and show a notable frequency in male patients. Histologically, gastroblastomas display a unique biphasic pattern, featuring both uniform spindle cells and nested epithelial cellular proliferation. A key distinguishing feature from GLI1-rearranged enteric tumors is their expression of keratins in the epithelial component, which serves as an important diagnostic marker. This distinct morphological profile has led to gastroblastoma’s recognition as a separate entity from GLI1-rearranged enteric tumors [29].

ACTB::GLI1 fusion, on the other hand, represents a different mechanism of *GLI1* oncogene activation. This fusion is characterized by the in-frame fusion between ACTB exon 3 and GLI1 exon 6 (Figure 1B). The strong ACTB promoter drives an increased expression of the fusion protein, leading to enhanced *GLI1* signaling. ACTB::GLI1 fusion has been observed in a distinct mesenchymal neoplasm exhibiting a pericystic phenotype, previously referred to as a t (7;12) translocation pericytoma, and has become an important diagnostic marker, helping differentiate tumors such as small round cell tumors, melanoma, and rhabdomyosarcomas [2,29]. ACTB::GLI1 tumors are characterized by uniform spindle cells that express immunoreactivity to SMA and laminin [16]. These lesions are predominantly benign and most commonly found in the tongue, with infrequent cases reported in the stomach and bone [5]. Despite their pericystic phenotype, tumors with t (7;12) are distinct from other pericystic tumors, such as myopericytoma and glomus tumors, which are associated with different genetic abnormalities, including *PDGFRB* mutations or *NOTCH1* gene rearrangements, respectively [15,30].

The NCOR2::GLI1, HNRNPA1::GLI1, and TUBA1B::GLI1 fusions, although less common, primarily affect gene expression and splicing mechanisms, potentially producing oncogenic protein variants and enhancing *GLI1*’s transcriptional activity, thereby promoting cancer cell proliferation, invasion, and modulation of inflammatory responses [31,32,33].

In cancers with PTCH1::GLI1 fusions, the normal tumor-suppressive function of PTCH1 is disrupted. PTCH1 typically inhibits the Smoothened (SMO) receptor, which, when activated by Hedgehog ligands, leads to the activation of *GLI1*. The fusion effectively bypasses this regulatory mechanism, resulting in continuous *GLI1* activation even in the absence of Hedgehog signaling [34]. This aberrant signaling can lead to increased tumor growth and recurrence, making it a potential target for therapeutic interventions [27]. Therefore, the presence of PTCH1::GLI1 fusions can serve as a biomarker for certain malignancies, influencing treatment decisions and prognostic assessments [35].

Another *GLI* fusion partner that drives oncogenic processes such as cell growth, survival, and invasion is the SYT::GLI1 fusion. SYT refers to the synaptotagmin family of proteins that can lead to aberrant activation of *GLI1,* which is particularly found in neuroendocrine tumors and glioblastomas [36,37]. SYT::GLI1 fusion may disrupt normal regulatory mechanisms, resulting in enhanced tumorigenesis and metastasis due to the dysregulation of target genes associated with these pathways, which can contribute to the development of resistance to therapies and complicating treatment strategies [38].

The precise molecular diagnosis of GLI1-rearranged enteric tumors necessitates an integrated approach utilizing multiple sophisticated diagnostic methodologies, with next-generation sequencing (NGS)-based fusion gene analysis serving as the cornerstone for comprehensive genome-wide assessments of genetic alterations (Figure 1A) [1]. Reverse transcriptase PCR (RT-PCR) complements the diagnosis by confirming the presence of fusion transcripts with high sensitivity and specificity and can therefore be used to validate the NGS result. Fluorescence in situ hybridization (FISH), using probes for ACTB, MALAT1, and GLI1, alongside dual-fusion FISH (D-FISH), offer a cost-effective alternative to detect the fusion of GLI1 loci and their partners (Figure 1A) [7]. FISH techniques can also be applied to distinguish *GLI* fusion tumors from other carcinomas and sarcomas that exhibit *MALAT1* rearrangement without *GLI1* involvement [39]. On the other hand, RNA-based and whole-transcriptome sequencing are helpful in demonstrating the expression of *GLI1* and its downstream targets, including *PTCH1*, *SOX2*, *VEGFA*, and *CCND1*, particularly when compared to normal gastric tissue [40]. Recent studies have revealed FOXS1 as a crucial GLI1-mediated target in breast cancer, with this molecule emerging as a central regulator of GLI1-driven cellular proliferation and tumor growth control, thereby expanding our understanding of the complex molecular networks underlying GLI-1 mediated neoplasms. These comprehensive molecular diagnostic approaches not only ensure accurate tumor classification but also provide valuable insights into disease mechanisms and potential therapeutic outcomes and ultimately support the development of more personalized and effective treatment strategies [40].

Understanding *GLI1*’s functional mechanisms not only enhances our investigations of the GLI1-rearranged enteric tumor mechanism but might also provide potential therapeutic targets for other tumors, paving the way for personalized treatment strategies. The *GLI1* gene encodes the zinc finger protein GLI1, also referred to as glioma-associated oncogene homolog 1, which is proven to have a potential role in the epithelial–mesenchymal transition through the induction of snail and repression of e-cadherin [41]. This protein functions as a downstream transcription factor within the Hedgehog (HH) signaling pathway, which is essential for processes such as DNA replication and the repair of DNA damage [42]. Aberrant activation of this pathway can result in the onset and progression of various cancers, such as medulloblastoma, rhabdomyosarcoma, basal cell carcinoma, glioblastoma, and numerous other solid tumors [43]. The canonical activation of the HH pathway begins when HH ligands, such as Sonic Hedgehog (SHH), bind to the transmembrane receptor Patched 1 (PTCH1), which relieves the inhibition of the G protein-coupled receptor SMO [44].

The HH signaling pathway can be categorized into three main types: Type I Noncanonical signaling, which functions through PTCH1 independently of SMO inhibition and traditional pathway components; Type II Noncanonical signaling, which involves SMO’s functions that do not depend on GLI1, primarily focusing on the activation of small GTPases and representing various SMO-dependent pathways; and Type III Noncanonical signaling, which includes pathways that activate the GLI1 transcription factor. Typically, the activation of the HH signaling pathway is dependent on the accurate transport of SMO to the primary cilium, a unique cell surface protrusion that acts as a crucial signaling center [45]. PTCH1 is strategically positioned near the base of the primary cilium, inhibiting the accumulation of SMO and influencing its activation potential. When SHH binds to PTCH1, PTCH1 is removed from the primary cilium, resulting in its destruction and enabling the translocation of SMO into the cilium [46,47]. In the absence of PTCH1, SMO undergoes phosphorylation and subsequent activation through its interaction with casein kinase 1 alpha (CK1α) and G protein-coupled receptor kinase 2 (GRK2), facilitating its relocation to the primary cilium via β-arrestin association, enabling the downstream activation of *GLI* transcription factors, and ultimately driving the expression of Hedgehog target genes [48].

In the type III pathway, the signaling operates independently of PTCH1-SMO signaling and corresponding methods for activating *GLI1,* known as alternative pathways [44,49]. GLI1 is translocated into the nucleus to activate target genes that undergo alternative splicing [50]. GLI1-producing variants like GLI1ΔN and tGLI1 act as gain-of-function transcription factors, sustaining the regulation of canonical *GLI1* target genes and inducing several carcinogenic abnormalities [51]. The interaction between SUFU and GLI1 is regulated through intricate mechanisms, including binding, sequestration in the cytoplasm, and dissociation. SUFU attaches to GLI1, keeping it in the cytoplasm and blocking its movement to the nucleus. When the pathway is activated, SUFU detaches from GLI1, enabling GLI1 to move into the nucleus and initiate the activation of target genes [52].

On the other hand, the intricate regulatory mechanisms of *GLI2* and *GLI3* are significantly different from *GLI1*. *GLI2* forms a direct complex with β-catenin, creating a crucial interaction that prevents the GSK-3β-mediated phosphorylation of β-catenin. This binding mechanism reduces β-catenin ubiquitination by 65%, consequently amplifying Wnt pathway signaling and promoting tumor growth. The significance of this interaction was demonstrated in xenograft models, where *GLI2* mutants lacking β-catenin-binding capability showed 40% reduced tumor growth and heightened chemotherapy sensitivity [53]. In contrast, the regulatory mechanism for *GLI3* is through its Ser1132 phosphorylation site. This site acts as a molecular switch, with CDK1-mediated phosphorylation during the G2/M phase determining *GLI*3’s function as either an activator or repressor [54]. These findings have opened new avenues for therapeutic intervention, suggesting that the dual targeting of these pathways might be more effective than single-agent approaches. Current drug development efforts are focusing on small molecule inhibitors that could either disrupt the *GLI2*-β-catenin interaction or modulate *GLI3*’s phosphorylation state, with several treatments showing promising results in preclinical trials [54,55].

Finally, it is important to note when interpreting the results of a molecular analysis that *GLI1* abnormalities are not limited to *GLI1*-rearranged enteric tumors. These molecular alterations have been identified across a remarkably diverse spectrum of malignancies, including central nervous system tumors (particularly gliomas), soft tissue sarcomas (notably alveolar rhabdomyosarcomas), bone tumors (such as chondrosarcomas), and various carcinomas. This widespread distribution highlights *GLI1*’s fundamental role in oncogenesis and cellular regulation across different tissue types [28].

## 4. Prognosis and Management of *GLI1*-Rearranged Enteric Tumors

*GLI1*-rearranged enteric tumors exhibit behaviors that are similar to those of low-grade to intermediate-grade sarcomas [1]. Currently reported cases of *GLI1* alterations with available clinical follow-up data indicate that approximately 38% of patients experienced locoregional recurrence or distant metastasis. Recurrence can arise following incomplete excision of the primary tumor, or there may be an extended interval of several years between the excision of the primary tumor and subsequent recurrence [56]. Elevated mitotic activity, tumor necrosis, and high-grade morphology are associated with unfavorable outcomes. Metastases have been observed in tumors that are characterized by low mitotic rates and the absence of necrosis, including those exhibiting a typical nested architecture and bland cytomorphology. Despite their potential for metastasis, patients who are diagnosed with GLI1-rearranged enteric tumors may experience survival durations ranging from 3 to 26 years [57].

Another meta-analysis examining 2847 patients across 15 studies demonstrated that high GLI1 expression correlates with a hazard ratio of 1.87 for overall survival in gastrointestinal cancers, establishing GLI1 as both a prognostic marker and therapeutic target [58]. However, the specific underlying molecular alteration, such as the *GLI1* fusion partner, does not appear to correlate with the prognosis [7]. Ultimately, additional data are needed to inform appropriate management for patients with *GLI1*-altered mesenchymal tumors [5].

Due to the malignant potential of *GLI1*-rearranged enteric tumors, surgical resection followed by surveillance CT of the abdomen and pelvis remains the primary standard of care. However, surgical management becomes a less viable treatment option in cases presenting with multiple lesions [1]. Alternative therapeutic approaches have been explored, including targeted molecular therapy focusing on the Hedgehog signaling pathway. Specifically, the inhibition of the SMO protein, a crucial component in Hedgehog signal transmission, has been investigated as a potential therapeutic strategy. However, recent studies have revealed that the GLI1 protein may develop resistance to SMO inhibitors, potentially limiting their therapeutic efficacy [59,60]. In addition, the relationship between GLI1 protein and the DNA damage repair protein NBS1 has been previously investigated in the context of chemotherapy-resistant colorectal cancer (CRC), which showed that elevated levels of both GLI1 and NBS1 strongly correlated with adverse clinical outcomes, including reduced survival rates and poor therapeutic response to 5-fluorouracil (5-FU) treatment [61]. The underlying mechanism demonstrated that GLI1 directly controls NBS1 expression, and blocking GLI1 effectively resulted in reducing NBS1 levels and therefore increasing cancer cells’ vulnerability to damage and death [62]. Furthermore, when GLI1 blockers were tested in laboratory models of BRAF-mutated colorectal cancer, they yielded promising results in reducing tumor growth by suppressing the NBS1 levels in BRAF-mutated cancers [63]. With the evolving landscape of cancer therapeutics, immunotherapy has emerged as a potential treatment alternative, particularly in cases where tumors express significant levels of PD-1/PD-L1 immune checkpoint proteins [64]. This immunotherapeutic approach may provide new opportunities for patients who have limited surgical options or have developed resistance to conventional treatments. Furthermore, the use of *GLI* inhibitors in combination with PARP inhibitors showed improvement in progression-free survival compared to PARP inhibitors alone in breast cancer cases, which highlighted the role of GLI signaling in conferring resistance to other treatments [65]. Therefore, the development of effective therapeutic strategies for *GLI1*-enteric tumors remains an active and dynamic area of research, emphasizing the need for personalized treatment approaches based on comprehensive molecular profiling and individual patient characteristics.

## 5. Summary

Our article provides a review of enteric tumors that have recently been identified as harboring the *GLI1* gene, which are associated with overlapping clinicopathological features that need to be differentiated from a broad range of other neoplasms, in particular myoepithelial tumors of soft tissue and glomus tumors. While MALAT1 and ACTB represent the primary fusion partners in GLI1-enteric tumors, the presence of these fusions is not disease-defining, as they also appear in plexiform fibromyxoma and gastroblastoma. Although these tumors typically follow an indolent course, they may carry significant risks when exceeding 5 cm in size or having a high-grade morphology. Accurate diagnosis requires advanced molecular diagnostic techniques, including next-generation sequencing, RT-PCR, FISH, and/or whole-transcriptome sequencing. Understanding the complex regulatory mechanisms of the signaling pathways associated with the HH pathway offers insights into possible *GLI1*-targeted treatments and the improvement of patient outcomes.

While surgical resection remains the standard of care, emerging therapeutic options such as immunotherapy and SMO inhibitors offer promising alternatives for treatment. This highlights the importance of personalized treatment strategies and ongoing research to optimize outcomes for patients with GLI1-associated tumors.

## Data Availability

No new data were created or analyzed in this study. Data sharing is not applicable to this article.
